# Insights into Adaptive Mechanisms of Extreme Acidophiles Based on Quorum Sensing/Quenching-Related Proteins

**DOI:** 10.1128/msystems.01491-21

**Published:** 2022-04-11

**Authors:** Shanshan Huang, Xueduan Liu, Weiyi Yang, Liyuan Ma, Huiying Li, Rui Liu, Jingxuan Qiu, Yiran Li

**Affiliations:** a School of Minerals Processing and Bioengineering, Central South University, Changsha, China; b Key Laboratory of Biometallurgy of Ministry of Education, Central South University, Changsha, China; c School of Environmental Studies, China University of Geosciences, Wuhan, China; University of Pretoria

**Keywords:** acidophiles, quorum sensing, quorum quenching, adaptive evolution, phylogeny

## Abstract

Quorum sensing (QS) is a unique mechanism for microorganisms to coordinate their activities through intercellular communication, including four main types of autoinducer-1 (AI-1, namely, *N*-acyl homoserine lactone [AHL]), AI-2, AI-3, and diffusible signaling factor [DSF]) based on signaling molecules. Quorum quenching (QQ) enzymes can disrupt the QS phenomenon by inactivating signaling molecules. QS is proposed to regulate biofilm formation in extremely acidic environments, but the QS/QQ-related genomic features in most acidophilic bacteria are still largely unknown. Here, genome annotation of 83 acidophiles from the genera *Acidithiobacillus*, *Leptospirillum*, *Sulfobacillus*, and *Acidiphilium* altogether revealed the existence of AI-1, AI-3, DSF, and AhlD (AHL degradation enzyme). The conservative investigation indicated that some QS/QQ-related proteins harbored key residues or motifs, which were necessary for their activities. Phylogenetic analysis showed that LuxI/R (AI-1 synthase/receptor), QseE/F (two-component system of AI-3), and RpfC/G (two-component system of DSF) exhibited similar evolutionary patterns within each pair. Meanwhile, proteins clustered approximately according to the species taxonomy. The widespread *Acidithiobacillus* strains, especially A. ferrooxidans, processed AI-1, AI-3, and DSF systems as well as the AhlD enzyme, which were favorable for their mutual information exchange and collective regulation of gene expression. Some members of the *Sulfobacillus* and *Acidiphilium* without AHL production capacity contained the AhlD enzyme, which may evolve for niche competition, while DSF in *Leptospirillum* and *Acidithiobacillus* could potentially combine with the cyclic diguanylate (c-di-GMP) pathway for self-defense and niche protection. This work will shed light on our understanding of the extent of communication networks and adaptive evolution among acidophiles via QS/QQ coping with environmental changes.

**IMPORTANCE** Understanding cell-cell communication QS is highly relevant for comprehending the regulatory and adaptive mechanisms among acidophiles in extremely acidic ecosystems. Previous studies focused on the existence and functionality of a single QS system in several acidophilic strains. Four representative genera were selected to decipher the distribution and role of QS and QQ integrated with the conservative and evolutionary analysis of related proteins. It was implicated that intra- or intersignaling circuits may work effectively based on different QS types to modulate biofilm formation and energy metabolism among acidophilic microbes. Some individuals could synthesize QQ enzymes for specific QS molecular inactivation to inhibit undesirable acidophile species. This study expanded our knowledge of the fundamental cognition and biological roles underlying the dynamical communication interactions among the coevolving acidophiles and provided a novel perspective for revealing their environmental adaptability.

## INTRODUCTION

Acidophiles, a category of primary extremophiles with unique features, are widely distributed in acid mine/rock drainage (AMD/ARD) environments (1). In these natural ecosystems, acidophilic microorganisms take active parts in the elemental cycle of sulfur and iron globally by oxidizing reduced inorganic sulfur compounds (RISCs) to sulfate and transforming ferrous and ferric ions (2, 3). It has been revealed that biofilm formation by attached bacterial cells is correlated with extracellular polymeric substance (EPS) production (4). As the prevalent and predominant genus thriving in AMD, the application of *Acidithiobacillus* in bioleaching has been studied extensively, as well as its adaptive evolution to extreme environments (5, 6). *Acidithiobacillus*, together with other acidophilic genera such as *Leptospirillum*, *Sulfobacillus* and *Acidiphilium*, exists in sulfide-bearing mineral environments between 20 and 40°C with pH lower than 3 (7, 8). Their attachment on ore surface and subsequent biofilm formation have been deciphered in great detail (9).

Quorum sensing (QS) is a sophisticated cell-to-cell communication process that enables bacteria to sense environmental changes (especially cell densities) and then orchestrate behaviors collectively, such as bioluminescence, motility, virulence factor production, and biofilm formation (10, 11). QS relies on the production, release, accumulation, and detection of extracellular signal molecules, called autoinducers (AIs). With the increasement of bacterial population density, AIs accumulate in the outer environment. Bacteria monitor the change of AI concentration, namely, the shift in cell amounts, and then jointly alter the expression levels of specific genes once the threshold has been reached (12). A novel conception called quorum quenching (QQ) enzymes, which inactivate QS molecules, has also emerged. QQ is termed a QS interference method, which is deemed to have evolved either by QS owners to eliminate excess signals or by other competitive organisms to attenuate their QS communication pathway (13).

Specifically, the autoinducer-1 (AI-1) system, composed of the canonical LuxI/LuxR pair, is one of the most well-studies QS systems in bacteria and is prevalent in Gram-negative (G^–^) bacteria such as *Proteobacteria* (14). LuxI catalyzes reactions between the homoserine lactone moiety contributor *S*-adenosylmethionine (SAM) and the acyl carrier protein (ACP) and principally synthesizes 3-oxo-hexanoyl homoserine lactone (OHHL), which is an *N*-acyl homoserine lactone (AHL) with the 3-oxo group. Then LuxR protein recognizes and binds to AHLs and consequently activates the expression of various QS-dependent genes (15). Chemical degradation of AHL compounds is a featured instance of QQ enzymes. AHL-lactonases and AHL-acylases have been described in several bacteria and developed as promising tools to block unnecessary gene expression and pathogenic phenotypes in medicine, aquaculture, and other fields (13). As a major type employed by both Gram-positive (G^+^) and G^−^ bacteria, the autoinducer-2 (AI-2) pathway serves in intra-species as well as inter-species communication modes (16). AI-2 is generated by the LuxS enzyme through a series of reactions and is recognized by three specific receptors, LuxP, LsrB, and RbsB (17). The autoinducer-3 (AI-3) is an amination product of aromatic compounds, as a less common interkingdom QS system, which is mainly found and elucidated in the enteric bacterium enterohemorrhagic Escherichia coli (EHEC). The QS E. coli regulators B and C (QseBC) in conjunction with E and F (QseEF) are key components of the AI-3/epinephrine (Epi)/norepinephrine (NE) signaling circuits. After binding with AI-3/Epi/NE, QseC auto-phosphorylates, and then it mediates the phosphorylation of QseB, thereby coordinating the expression of flagellar, motility, and virulence genes (18). QseEF share a similar mechanism but have a narrower distribution and play essential roles in the regulation of attaching and effacing (AE) lesion formation (18, 19). The diffusible signaling factor (DSF) represents a novel kind of QS system, which is exemplified in Xanthomonas campestris pv. *campestris*. DSF family signals synthesized by RpfF are transduced by the sensor protein RpfC to its receptor RpfG, to regulate the expression of DSF-controlled genes based on the signaling cascade encompassing RpfB, cyclic diguanylate (c-di-GMP), and Clp in X. campestris pv. *campestris* (20).

Bioinformatic prediction and experimental validation have determined the existence of QS signaling communication within acidophiles. For example, it is well known that Acidithiobacillus ferrooxidans possesses a functional AHL-type QS. The system has evolved unique regulatory patterns specific to the energy substrates, which expands our understanding of AI-1 to adjust the gene transcription of *A. ferrooxidans* for cell growth and population development in Fe/S-enriched extremely acidic environments (21, 22). It is known that several *A. ferrooxidans* and Acidithiobacillus thiooxidans strains synthesize AHLs, which could certainly exert functions within the same species or might make *A. ferrooxidans*/A. thiooxidans communication happen. Although two Leptospirillum ferrooxidans strains, DSMZ 2391 and DSMZ 2705, could not produce AHL, they can sense external AHLs secreted by other microbes located in its ecological niche by expressing a SdiA-like protein, just like the case of Escherichia and Salmonella (23). After addition of exogenous DSF, the attached cells of Acidithiobacillus caldus, Leptospirillum ferriphilum, and Sulfobacillus thermosulfidooxidans on mineral surfaces decline obviously, so the *Leptospirillum* spp. are proved to have the ability to produce the DSF family compounds (24). It will be fascinating to examine the effects of DSF in mixed cultures involving *Acidithiobacillus*, *Leptospirillum*, and heterotrophic *Acidiphilium* spp. in the future (24).

Hence, QS are powerful mediators of intra- to inter-species communication circuitry, and some acidophilic members maintain complex interactions with others by this approach. With the increasing development of sequencing techniques, more and more genomic data of acidophiles are readily available. In this study, we reported the bioinformatic survey of QS or QQ in the four above-mentioned acidophilic genera and focused on the AI-1 system being inspected frequently. With regard to the qualified QS/QQ-related proteins, the sequence alignment was conducted to check key residues or motifs for authentication validity. Then phylogenetic analysis was carried out to determine evolutionary relationships. Finally, we deciphered the system distribution properties and assessed our current understanding of QS in acidophiles, aiming to offer genomic evidence for its potential role and function in extreme AMD ecosystems.

## RESULTS

### Overview of the QS/QQ-related protein distribution in acidophiles.

The genome assembly plus annotation statistics of 83 acidophiles belonging to 4 genera together with their basic features are presented in Table S1. There were 77 kinds of KEGG Orthology (KO) annotated to “quorum sensing” (ko02024) based on the KEGG database in these strains. Accordingly, the QS-related protein entries were explored in detail and their potential functions are exhibited in Table 1.

**TABLE 1:** General information of annotated KO related to QS in 83 acidophiles

KEGG Orthology number	Protein	Domain	Definition
K01580	GadA/B	COG0076	Glutamate decarboxylase (EC 4.1.1.15)
K01657	TrpE	COG0147	Anthranilate synthase component I [EC:4.1.3.27]
K01658	TrpG	COG0512	Anthranilate synthase component II (EC 4.1.3.27)
K01897	RpfB	COG0318, COG1022	Long-chain acyl-CoA synthetase (EC 6.2.1.3)
K02031	DdpD	COG0444, COG1123	Peptide/nickel transport system ATP-binding protein
K02033	ABC.PE.P	COG0601	Peptide/nickel transport system permease protein
K02034	ABC.PE.P1	COG1173	Peptide/nickel transport system permease protein
K02035	ABC.PE.S	COG0747	Peptide/nickel transport system substrate-binding protein
K03070	SecA	COG0653	Preprotein translocase subunit SecA (EC 7.4.2.8)
K03071	SecB	COG1952	Preprotein translocase subunit SecB
K03073	SecE	COG0690	Preprotein translocase subunit SecE
K03075	SecG	COG1314	Preprotein translocase subunit SecG
K03076	SecY	COG0201	Preprotein translocase subunit SecY
K03106	SRP54	COG0541	Signal recognition particle subunit SRP54 (EC 3.6.5.4)
K03110	FtsY	COG0552	Fused signal recognition particle receptor
K03210	YajC	COG1862	Preprotein translocase subunit YajC
K03217	YidC	COG0706	YidC/Oxa1 family membrane protein insertase
K10823	OppF	COG4608	Oligopeptide transport system ATP-binding protein
K10914	Clp	COG0664	CRP/FNR family transcriptional regulator, cyclic AMP receptor protein
K11752	RibD	COG0117, COG1985	Diaminohydroxy phosphoribosyl aminopyrimidine deaminase/5-amino-6-(5-phosphoribosylamino) uracil reductase (EC 3.5.4.26 1.1.1.193)
K01114	Plc	COG1923	Phospholipase C (EC 3.1.4.3)
K03666	Hfq	COG3512	Host factor-I protein
K07667	KdpE	COG0745	Two-component system, OmpR family, KDP operon response regulator KdpE
K13075	AhlD	COG0491	*N*-acyl homoserine lactone hydrolase (EC 3.1.1.81)
K20527	TrbB	COG4962	Type IV secretion system protein TrbB (EC 7.4.2.8)
K20528	TrbC	COG3838	Type IV secretion system protein TrbC
K20529	TrbD	COG5268	Type IV secretion system protein TrbD
K20530	TrbE	COG4962	Type IV secretion system protein TrbE (EC 7.4.2.8)
K20531	TrbF	COG3701	Type IV secretion system protein TrbF
K20532	TrbG	COG3504	Type IV secretion system protein TrbG
K20533	TrbI	COG2948	Type IV secretion system protein TrbI
K07344	TrbL	COG3846	Type IV secretion system protein TrbL
K01497	RibA	COG0807	GTP cyclohydrolase II (EC 3.5.4.25)
K01995	LivG	COG0411	Branched-chain amino acid transport system ATP-binding protein
K01996	LivF	COG0410	Branched-chain amino acid transport system ATP-binding protein
K01997	LivH	COG0559	Branched-chain amino acid transport system permease protein
K01998	LivM	COG4177	Branched-chain amino acid transport system permease protein
K01999	LivK	COG0683	Branched-chain amino acid transport system substrate-binding protein
K02032	DdpF	COG1123, COG1124	Peptide/nickel transport system ATP-binding protein
K02052	ABC.SP.A	COG3842	Putative spermidine/putrescine transport system ATP-binding protein
K02053	ABC.SP.P	COG1177	Putative spermidine/putrescine transport system permease protein
K02054	ABC.SP.P2	COG1176	Putative spermidine/putrescine transport system permease protein
K02055	ABC.SP.S	COG0687	Putative spermidine/putrescine transport system substrate-binding protein
K06998	PhzF	COG0384	Trans-2,3-dihydro-3-hydroxyanthranilate isomerase (EC 5.3.3.17)
K07699	Spo0A	COG0784	Two-component system, response regulator, stage 0 sporulation protein A
K20327	XagB	COG1215	Glycosyltransferase XagB
K09936	ToxF	COG3238	Bacterial/archaeal transporter family-2 protein
K15580	OppA	COG4166	Oligopeptide transport system substrate-binding protein
K18139	ToxI	COG1538	Outer membrane protein, multidrug efflux system
K20265	GadC	COG0531	Glutamate:GABA antiporter
K20266	TrbJ	COG5314	Type IV secretion system protein TrbJ
K07666	QseB	COG0745	Two-component system, OmpR family, response regulator
K10715	RpfC	COG0642, COG0784	Two-component system, sensor histidine kinase RpfC (EC 2.7.13.3)
K13815	RpfG	COG3437	Two-component system, response regulator RpfG
K13816	RpfF	COG1024	DSF synthase
K01626	PhzC	COG0722	3-Deoxy-7-phosphoheptulonate synthase (EC 2.5.1.54)
K09823	Zur	COG0735	Fur family transcriptional regulator, zinc uptake regulator
K10555	LsrB	COG1879	AI-2 transport system substrate-binding protein
K20249	RaiI	COG3916	Acyl homoserine lactone synthase
K20326	XagA		Protein XagA
K02490	Spo0F	COG0784	Two-component system, response regulator, stage 0 sporulation protein F (EC 2.3.1.184)
K07692	DegU	COG2197	Two-component system, NarL family, response regulator DegU
K14982	CiaH	COG0642	Two-component system, OmpR family, sensor histidine kinase CiaH (EC 2.7.13.3)
K15583	OppD	COG0444	Oligopeptide transport system ATP-binding protein
K20332	ToxC	COG2319	Toxoflavin biosynthesis protein ToxC
K20333	ToxD	COG1262	Toxoflavin biosynthesis protein ToxD
K15581	OppB	COG0601	Oligopeptide transport system permease protein
K15582	OppC	COG1173	Oligopeptide transport system permease protein
K20334	CviR	COG2771, COG2197	LuxR family transcriptional regulator, quorum-sensing system regulator CviR
K02402	FlhC		Flagellar transcriptional activator FlhC
K07711	QseE	COG0642	Two-component system, NtrC family, sensor histidine kinase GlrK (EC 2.7.13.3)
K07715	QseF	COG2204	Two-component system, NtrC family, response regulator GlrR
K13061	RhlI	COG3916	Acyl homoserine lactone synthase (EC 2.3.1.184)
K18100	RhlA	COG0596	Rhamnosyltransferase subunit A (EC 2.4.1.–)
K18101	RhlB	COG1819	Rhamnosyltransferase subunit B (EC 2.4.1.–)
K12990	RhlC	COG1216	Rhamnosyltransferase (EC 2.4.1.–)
K18306	ToxG	COG0845	Membrane fusion protein, multidrug efflux system

10.1128/msystems.01491-21.6 TABLE S1 Main features and QS-related annotation of the 83 acidophiles. Download Table S1, DOCX file, 0.03 MB. Copyright © 2022 Huang et al. 2022 Huang et al. https://creativecommons.org/licenses/by/4.0/ This content is distributed under the terms of the Creative Commons Attribution 4.0 International license.

A considerable number of KOs (19; ∼25%) were shared by four acidophilic genera (Fig. S1). Among them, several (SecA, KO number K03070; SecE, K03073; SecG, K03075; SecY, K03076; SRP54, K03106; FtsY, K03110; YajC, K03210; YidC, K03217) were the part of general secretory (Sec) pathway of the type II secretion system (T2SS), which could be regulated by the RhlI/R-QS and DSF-QS systems (25). In addition, PhnA (K01657) and PhnB (K01658) were harbored widely, and the enzymes encoded by them participate in the synthesis of a typical Pseudomonas quinolone signal (PQS), which is 2-heptyl-3-hydroxy-4(1H)-quinolone, another type of QS signal. Notably, RpfB (K01897) and Clp (K10914), constituents of the DSF-type QS system (26), were found to be inclusive in these genera.

10.1128/msystems.01491-21.1 FIG S1 Venn diagram of annotated KO distribution between strains in the genera *Acidithiobacillus*, *Leptospirillum*, *Sulfobacillus*, and *Acidiphilium*. Download FIG S1, TIF file, 0.1 MB. Copyright © 2022 Huang et al. 2022 Huang et al. https://creativecommons.org/licenses/by/4.0/ This content is distributed under the terms of the Creative Commons Attribution 4.0 International license.

Additionally, some exclusive examples deserved attention in particular individual strains. As a response regulator, QseB (K07666) combined with sensor kinase, QseC, comprising QseBC two-component QS signaling. QseB was only detected in Acidithiobacillus sulfuriphilus CJ-2, Sulfobacillus acidophilus DSM 10332, and S. acidophilus TPY, but QseC was not observed in this study. It was presumed that the solo QseB might be capable of activating FlhC (KO number K02402), responsible for flagella and motility in *A. sulfuriphilus* CJ-2, since QseB potentially possessed dual regulatory capacities in the absence of QseC (27), which remains to be characterized. LsrB (KO number K10555) was only discovered in Acidiphilium angustum ATCC 35903, Acidiphilium rubrum ATCC 35905, and *Acidiphilium* sp. strain 34-64-41, which is a part of the LsrACDB transporter, accountable for importing extracellular AI-2. Due to the lack of other transporters, whether LsrB worked effectively is still doubtful in these strains.

### Characterization of diverse QS/QQ systems among acidophiles.

**Sequence, phylogeny, and structure analysis of LuxI/R homologs identified in acidophiles.** In the archetypical AHL-QS system, a LuxI and a LuxR are always located adjacent to each other. In addition to the canonical LuxI/R pair, extra LuxR regulators that are not in the vicinity of a cognate LuxI are frequently observed, termed solos/orphans (28). Likewise, LuxI solos could be defined as well (29). In this report, one putative LuxI/R homolog pair (RhlI and SolR) has been identified in *A. ferrooxidans* (8 strains except BY0502), A. thiooxidans (9 strains except ATCC 19377 and Licanantay), Acidithiobacillus ferridurans IO-2C & JCM 18981, *Acidithiobacillus* sp. strain ‘AMD consortium’ and *Acidithiobacillus* sp. strain GGI-221. Also, an additional RhlI solo has been detected in *A. ferrooxidans* BY-3. Intriguingly, a LuxI homolog (RaiI) has been found alone without the presence of any LuxR homologues in *Acidiphilium* sp. strain 37-67-22. The LuxR homolog CviR (KO number K20334) was carried only by L. ferrooxidans C2-3, also apparently the only one referring to AI-1 QS system in *Leptospirillum*. These LuxI/R homolog proteins were found to contain their corresponding signature InterPro domains, except for RaiI, which lacks the autoinducer synthesis conserved site, IPR018311. Since a recent study has demonstrated that LuxI proteins of *Pandoraea* species could work properly despite the absence of IPR018311 (30), we further investigated the protein RaiI.

**FIG 1:**
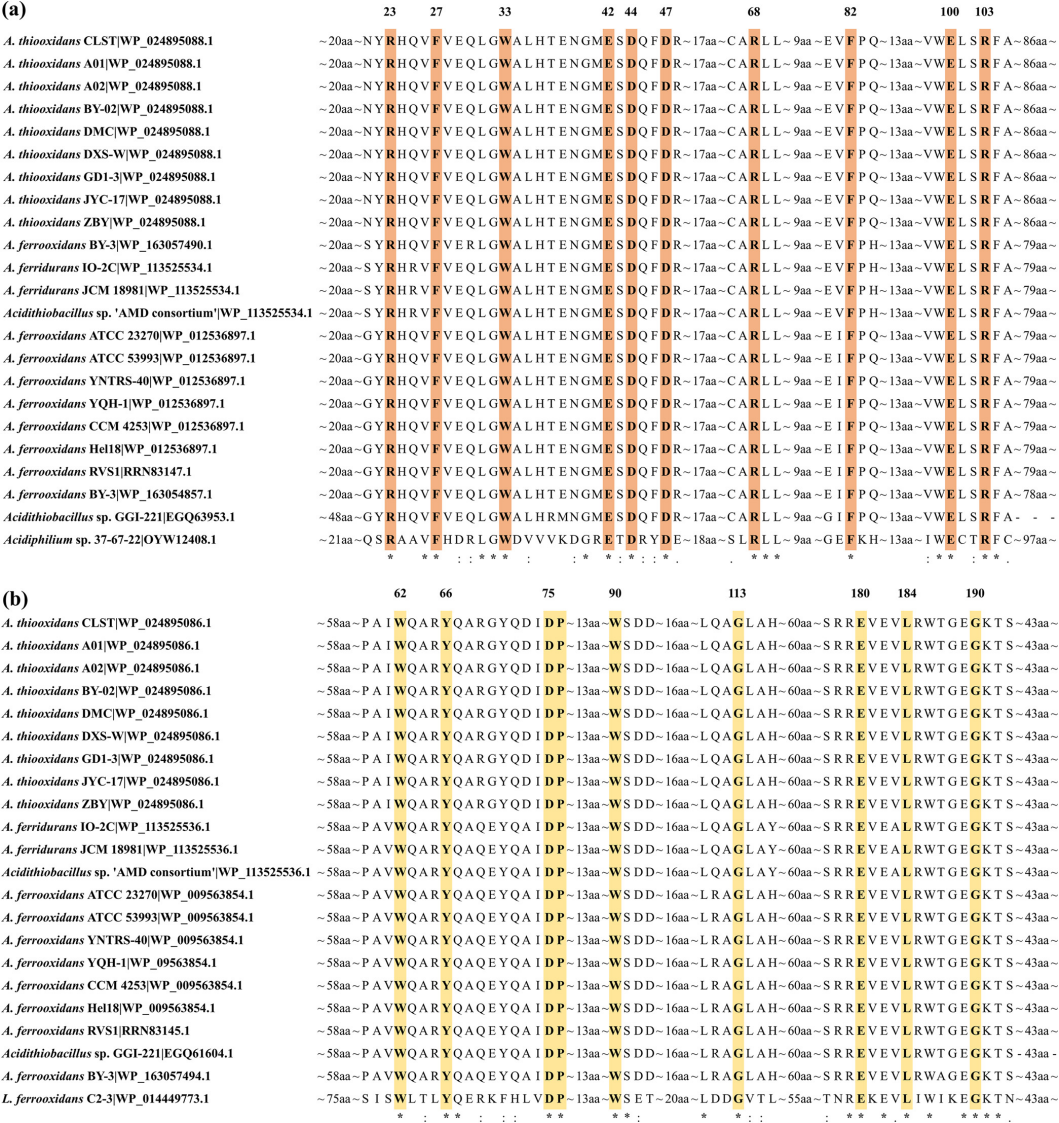
(a and b) Sequence alignments of RhlI in *Acidithiobacillus* strains and RaiI in an *Acidiphilium* strain (a) and SolR in *Acidithiobacillus* strains and CviR in a *Leptospirillum* strain (b) and the conservation of key residues. Residues are highlighted and numbered based on the protein sequence of the first line.

As shown in Fig. 1a, multiple-alignment analysis of LuxI homolog sequences revealed a consistent profile of critical residues R23, F27, W33, E42, D44, D47, and R68, which were within the supposed active-site region, and F82, E100, and R103 in the area essential for conformation of the active site. In the case of LuxR homologs, key conserved residues (W62, Y66, D75, P76, W90, and G113) are signature amino acids in the autoinducer-binding domain, and E180, L184, and G190 are three essential amino acids in the DNA-binding domain (Fig. 1b).

**FIG 2:**
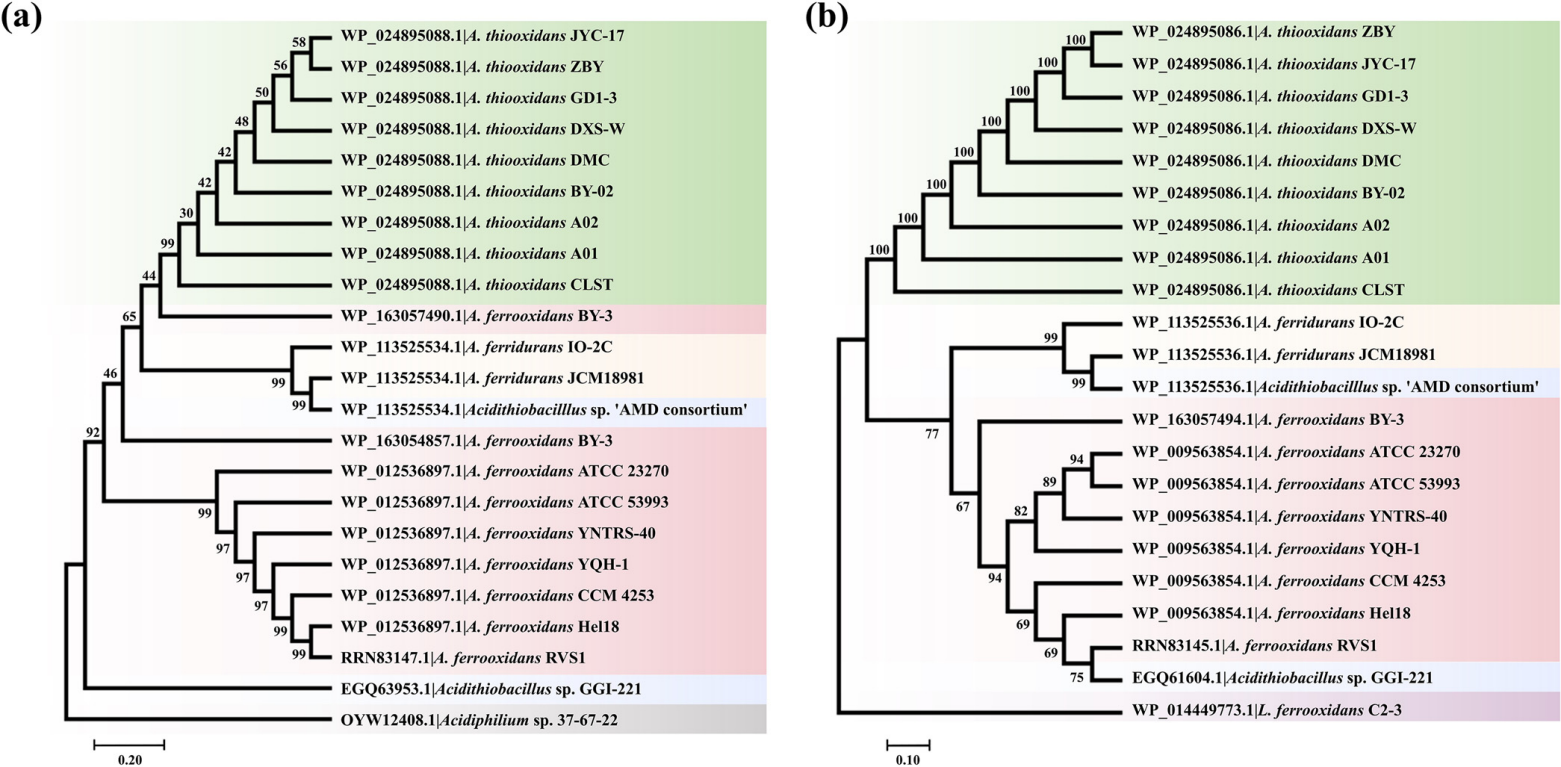
(a and b) Phylogenetic analysis of RhlI in an *Acidithiobacillus* strains and RaiI in an *Acidiphilium* strain (a) and SolR in *Acidithiobacillus* strains and CviR in a *Leptospirillum* strain (b). The trees were constructed using the neighbor-joining method with a bootstrap value of 5,000. Colors indicate different bacterial species.

Phylogenetic trees were constructed to further investigate the relatedness of LuxI and LuxR homologs. Proteins from the same species were generally clustered together (Fig. 2), forming two major branches composed of candidates from A. thiooxidans and *A. ferrooxidans*, respectively. Not surprisingly, RaiI of *Acidiphilium* and CviR of *Leptospirillum* were separated from their homologues, representing a distinct clade at the bottom of each tree. Furthermore, proteins of *Acidithiobacillus* sp. ‘AMD consortium’ were grouped together with those from two *A. ferridurans* strains; their RhlIs were clustered with clade A. thiooxidans (Fig. 2a), while their SolRs were located closer to the *A. ferrooxidans* clade (Fig. 2b). Although the RhlIs of *A. ferrooxidans* BY-3 were distinctive from each other, they grouped with the A. thiooxidans branch on the whole; in comparison, its SolR was clustered into the branch *A. ferrooxidans*, suggesting that they may have evolved from different ancestors.

**FIG 3:**
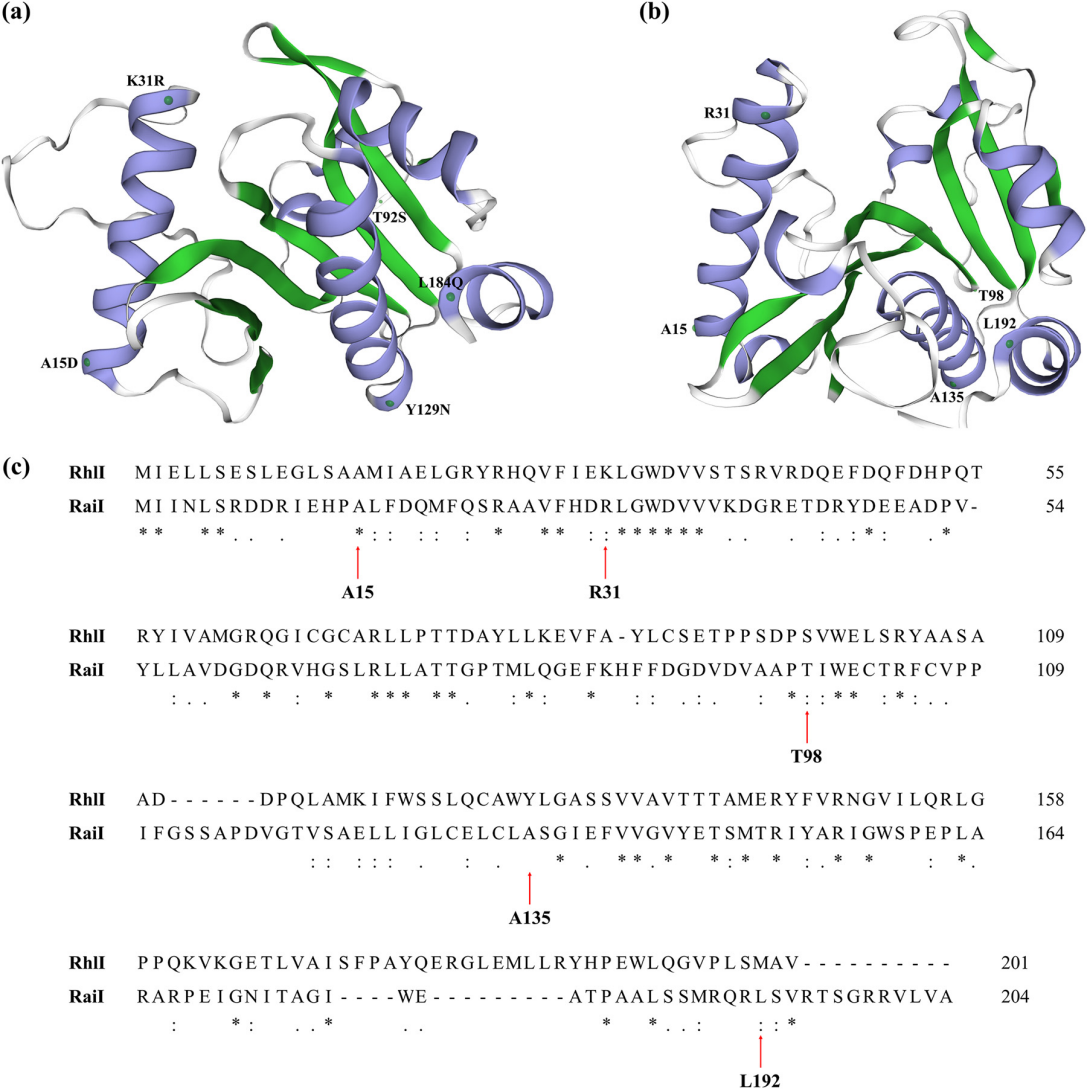
(a to c) Locations of the mutations in the RhlI (31) homologous models (a), RaiI models of *Acidiphilium* sp. 37-67-22 (b), and sequence alignments of RhlI and RaiI (c).

By directed enzyme evolution, the RhlI-4M1 mutant of Pseudomonas aeruginosa was generated and discovered to contain five amino acid substitutions crucial for its increased activities (31). We created a phylogenetic tree of this protein sequence with LuxI homologs in the present study; intriguingly, it was clustered with RaiI of *Acidiphilium* sp. 37-67-22 (Fig. S2). Hence, crystal structures of RhlI-4M1 and RaiI were established coupling with sequence alignments to show the scattered mutant sites (Fig. 3). The substitution K31R (R31 in RaiI) is likely to mediate the interactions with the hexanoyl sidechain. In addition, the K31R occurred at the pocket that is greatly specific for the octanoyl chain. A substitution of threonine with a similar amino acid serine was located at site 92 (Fig. 3a), which corresponded to T98 in RaiI (Fig. 3b), indicating that a hydrophilic amino acid residue is favored for long-chain AHLs at this position. As for the substitution of L with Q at position 184 (Fig. 3a), it matched L192 in RaiI (Fig. 3b), suggesting that a hydrophilic amino acid residue is preferred for OHHL at this loop.

10.1128/msystems.01491-21.2 FIG S2 Phylogenetic analysis of RhlI in *Acidithiobacillus* strains and RaiI in an *Acidiphilium* strain and RhlI-4M1 mutant. The trees were constructed using the neighbor-joining method with a bootstrap value of 5,000. The red rectangle indicates that RaiI and RhlI-4M1 were clustered closer. Download FIG S2, TIF file, 2.0 MB. Copyright © 2022 Huang et al. 2022 Huang et al. https://creativecommons.org/licenses/by/4.0/ This content is distributed under the terms of the Creative Commons Attribution 4.0 International license.

**FIG 4:**
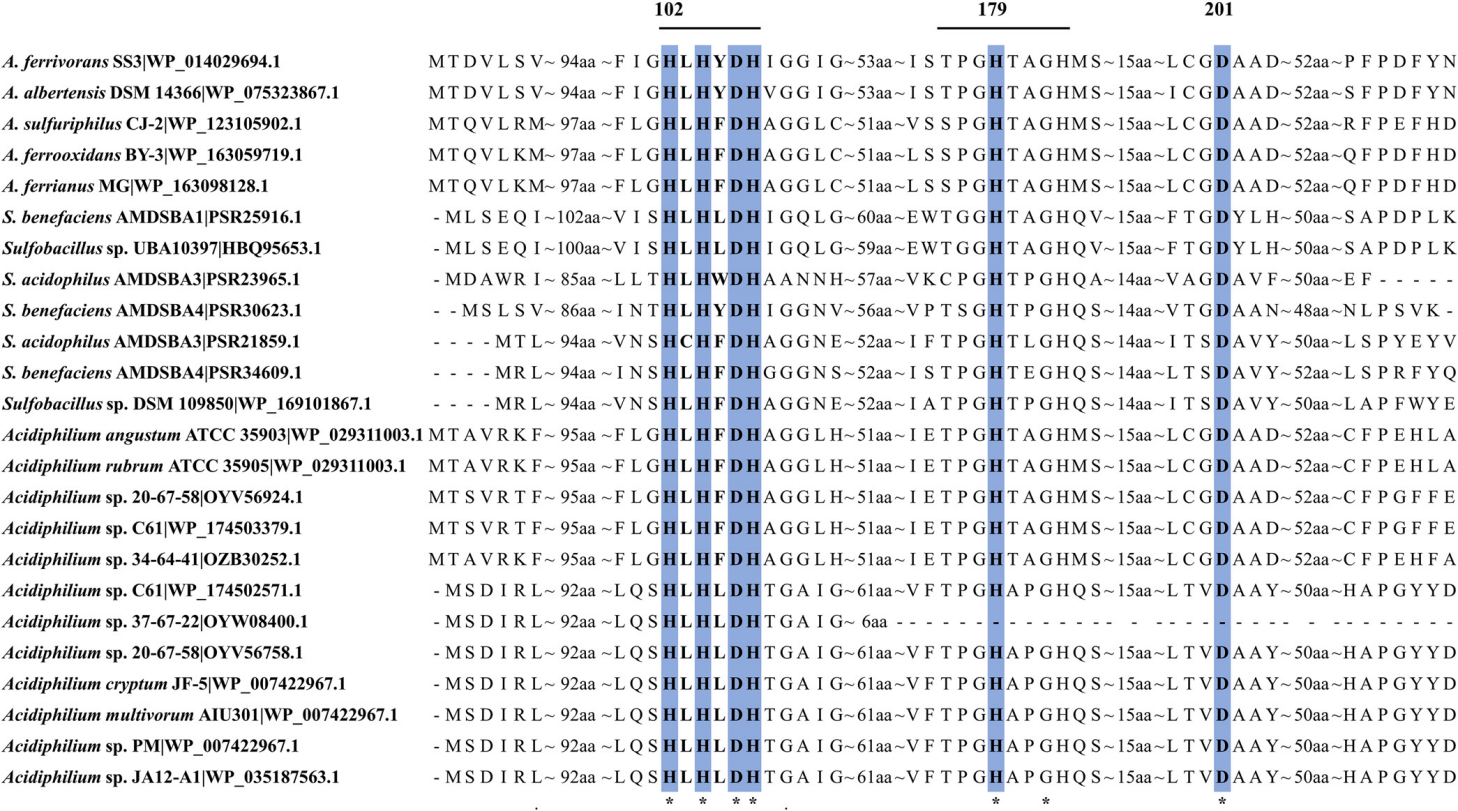
Sequence alignments of AhlD. Horizontal lines denote conserved ^102^HXHXDH^107^ and ^176^TPGHTPGH^183^ motifs necessary for AHLase activity. The important conserved residues are highlighted and numbered based on the protein sequence of the first line.

**Distribution and sequence comparison of AhlD detected among acidophiles.** AhlD has been reported to be an AHL-degrading enzyme that hydrolyzes the ester bond of the homoserine lactone ring of AHLs (32). It could be observed that AhlD was relatively widespread according to the bioinformatic prediction, distributed in 5 *Acidithiobacillus* strains belonging to different species, 5 *Sulfobacillus* strains, and 10 *Acidiphilium* strains. In addition, two AhlDs were identified in strains S. acidophilus AMDSBA3, Sulfobacillus benefaciens AMDSBA4, *Acidiphilium* sp. 20-67-58, and *Acidiphilium* sp. C61. The sequence analysis showed that these AhlDs displayed a highly conserved ^102^HXHXDH-63 amino acids-^179^H-24 amino acids-^201^D pattern and ^176^TPGHTPGH^183^ motif with relatively lower conservatism (Fig. 4), which has been proved to be critical for AHLase activity in its enzyme family, such as AiiA, AiiB, and QqlR. Furthermore, the HXHXDH≈H feature is considered the zinc metallo-hydrolase criterion, implying that AhlD might actually belong to the zinc metallohydrolase family (33). While the AhlD of *Acidiphilium* sp. 37-67-22 lacked the key motif and one residue due to the limited length, its functional activity was questionable.

**Distribution and phylogenetic analysis of QseE and QseF in acidophiles.** QseEF is part of the AI-3/epinephrine/norepinephrine signaling system, which is considered a cognate two-component system. QseE is a histidine kinase, which could phosphorylate the response regulator QseF by interacting with signaling molecules. QseE and QseF have been widely detected in 41 *Acidithiobacillus* strains. Furthermore, the majority of them harbored two QseEF pairs. By comparison, strains A02 and JYC-17 of A. thiooxidans had an extra QseF and Acidithiobacillus ferrivorans 21-59-9 lacked a QseE. In addition, *A. caldus* S1 possessed only one QseF without a cognate sensor kinase. Then, these 79 QseE protein sequences and 83 QseF protein sequences were included in the construction of evolutionary trees, which showcased similar clustering features (Fig. S3). In general, two QseEs or QseFs from the same strain were apart from each other, forming two almost symmetrical clusters in each circle tree, except that both QseF proteins of *Acidithiobacillus* sp. GGI-221 were on the right side (Fig. S3b). The separate clustered QseE or QseFs shared comparable distribution properties for which proteins from the same species were obviously grouped together. In addition, proteins of *A. ferridurans* strains JCM 18981 and IO-2C, *Acidithiobacillus* sp. ‘AMD consortium’ and *Acidithiobacillus* sp. GGI-221 were located in the *A. ferrooxidans* branch, while protein entries of Acidithiobacillus albertensis DSM 14366 were clustered in the A. thiooxidans clade. Surprisingly, proteins from *A. ferrooxidans* BY0502 were located in the group *A. ferrivorans*. In short, QseE/F proteins detected in same *Acidithiobacillus* species exhibited similar evolutionary tendencies, and most strains may possess two sets of QseEF system with different homologous relationships.

10.1128/msystems.01491-21.3 FIG S3 (a and b) Phylogenetic analysis of QseE (a) and QseF (b) proteins identified in *Acidithiobacillus* strains. The trees were constructed using the neighbor-joining method with bootstrap values of 5,000 (a) and 4,090 (b) since 910 replicates failed. Sequences for the following strains were removed from the tree of panel b because no common sites were found for them: *A. ferrivorans* 21-59-9 (GenBank version number OYV82609.1), A. thiooxidans A02 (WP_065968265.1 and WP_139110658.1) and A. thiooxidans JYC-17 (WP_139113708.1). Colors indicate different bacterial species. Download FIG S3, TIF file, 2.3 MB. Copyright © 2022 Huang et al. 2022 Huang et al. https://creativecommons.org/licenses/by/4.0/ This content is distributed under the terms of the Creative Commons Attribution 4.0 International license.

**Profile of a DSF type-based QS system among acidophiles.** The typical category of DSF-QS system is composed of RpfC, RpfG, RpfF (in the *rpf* gene cluster), RpfB, and Clp, which has been inspected in a wide range of bacterial genomes and viewed as a conserved mechanism (20). In this study, the *Leptospirillum* genomes were annotated to contain this kind of QS system even though L. ferriphilum ZJ lost its RpfF. In addition, *A. ferrivorans* (all 7 strains), A. thiooxidans (8 strains except ATCC 19377, CLST, and Licanantay), *A. ferrooxidans* BY0502, and *A. ferridurans* IO-2C harbored a complete DSF-type signaling system, and so did *A. albertensis* DSM 14366, which lacked the Clp regulator. Then these genomes were used in the following analysis.

RpfB was predicted to encode a fatty acyl-CoA ligase (FCL; EC 6.2.1.3), which was identified in all the genomes except *Acidiphilium* sp. strain 21-66-27 in the current report. By the sequence alignments (Fig. S4a), we assumed that the RpfBs had the ATP/AMP-binding motif as well as active-site threonine and glutamate residues crucial for catalytic activity. Moreover, except for one of three RpfBs of *A. ferrivorans* 21-59-9 and RpfBs from the *A. ferrivorans* PRJEB5721 and CF27 strains, the signature FCL motif was also identified in vast RpfBs, proving its authentic and vital roles in fatty acid β-oxidation, which has been reported in X. campestris pv. *campestris* (34). As for DSF synthase RpfF, it conserved the amino acids G86, G138, responsible for the thioesterase activity, acidic E141, and E161, critical for catalysis (35) as shown in Fig. S4b.

10.1128/msystems.01491-21.4 FIG S4 (a and b) Sequence alignments of RpfB (a) indicating the conservation of ATP/AMP and FCL motifs and RpfF (b). The important conserved residues are highlighted and numbered based on the protein sequence of the first line. Download FIG S4, TIF file, 2.3 MB. Copyright © 2022 Huang et al. 2022 Huang et al. https://creativecommons.org/licenses/by/4.0/ This content is distributed under the terms of the Creative Commons Attribution 4.0 International license.

**FIG 5:**
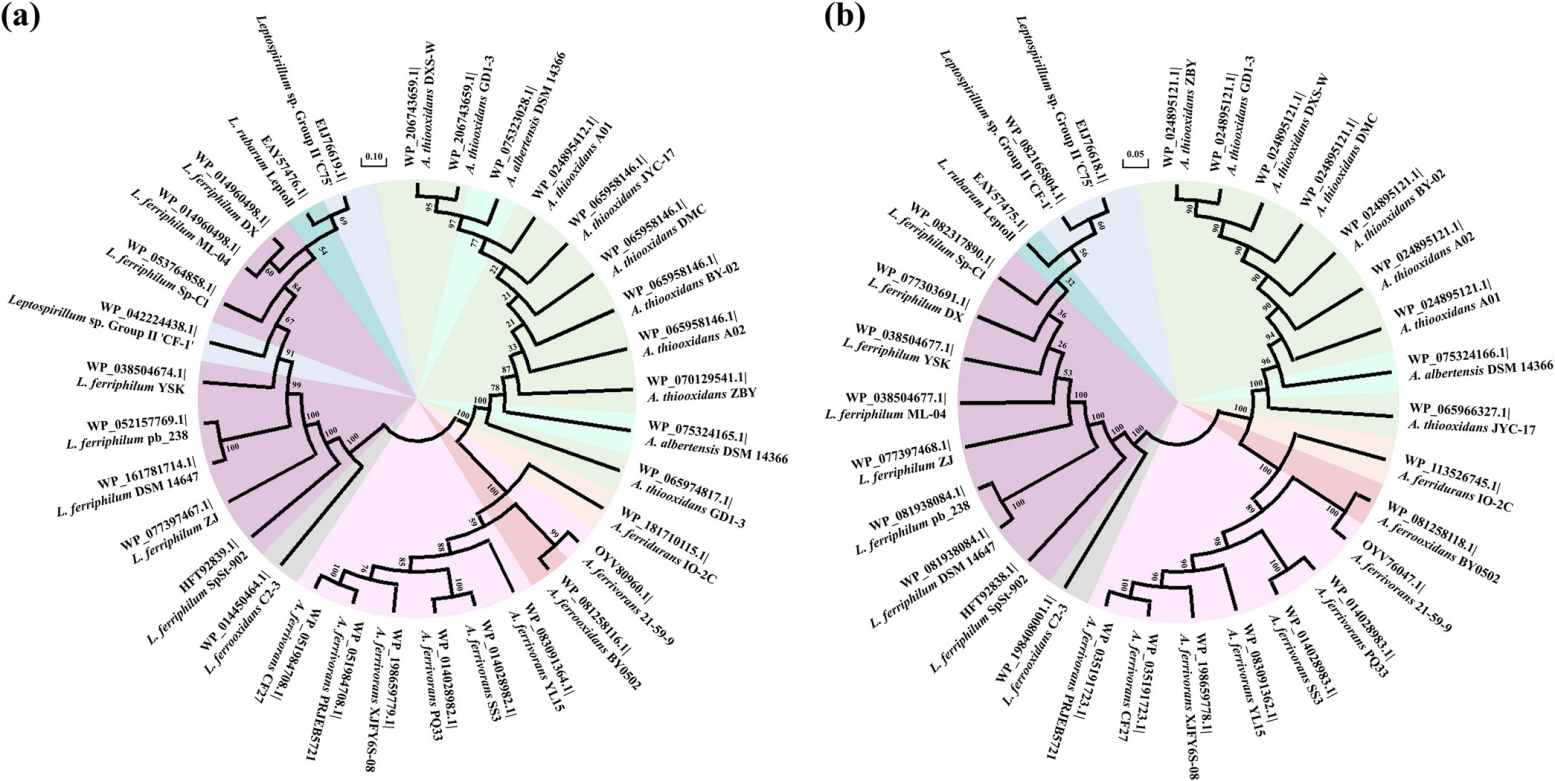
(a and b) Phylogenetic analysis of RpfC (a) and RpfG (b) proteins identified in *Acidithiobacillus* and *Leptospirillum* strains. The trees were constructed using the neighbor-joining method with a bootstrap value of 4,318 (a), since 682 replicates failed, and 5,000 (b). Sequences for the following strains were removed from tree of panel a because no common sites were found for them: Leptospirillum ferriphilium DSM 14647 (GenBank version number WP_036081472.1), A. thiooxidans DXS-W (WP_065974817.1), and *L. ferriphilium* pb_238 (WP_036081472.1). Colors indicate different bacterial species.

The sensor kinase RpfC and the response regulator RpfG constitute a two-component system involved in the perception and transduction of DSF family signals. One RpfG was detected in each strain, whereas 5 genomes carried two distinct RpfCs. Phylogenetic analysis was conducted with some deletion then. Protein entries from *Acidithiobacillus* and *Leptospirillum* were separated and formed different clusters, mainly *Leptospirillum*, *A. ferrivorans*, and A. thiooxidans (Fig. 5). RpfC/G of L. ferriphilum SpSt-902 and L. ferrooxidans C2-3 were always parted as single outgroups, indicating their different evolutionary characteristics with other *Leptospirillum*. Additionally, proteins of *A. ferrooxidans* BY0502 and *A. ferridurans* IO-2C were located in the *A. ferrivorans* branch; meanwhile, proteins of *A. albertensis* DSM 14366 were grouped within clade A. thiooxidans, sharing some common evolutionary features to a certain extent.

### The gene neighborhoods around LuxI/R homologs of acidophiles.

**FIG 6:**
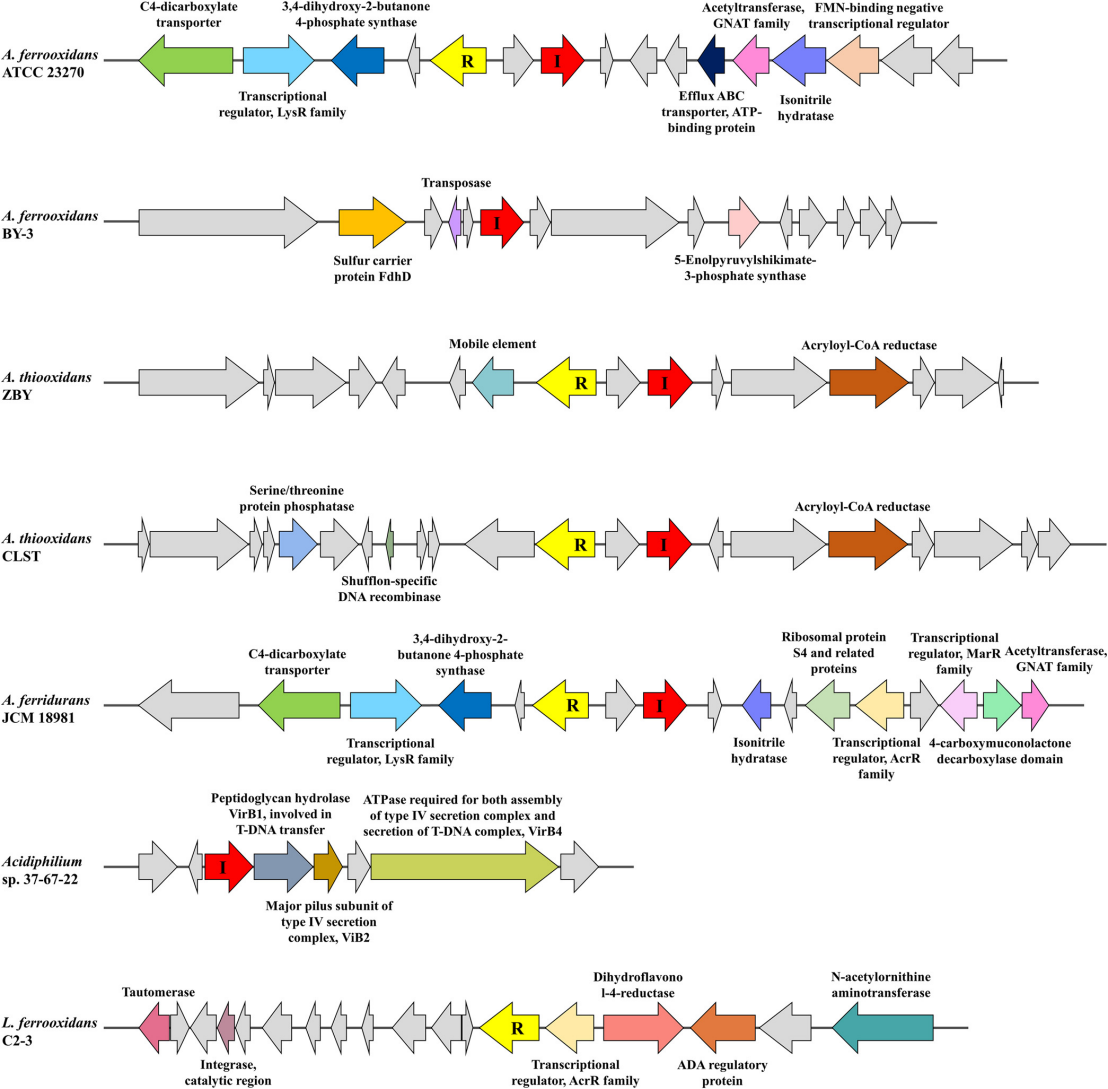
The arrangement of identified LuxI and LuxR homologs and the 5-kbp genetic region surrounding selected acidophiles. Genes and their orientations are depicted with arrows using the following colors: red, LuxI homolog; yellow, LuxR homolog; gray, hypothetical protein.

It is expected that neighboring genes are targeted for the transcriptional regulators in bacteria. LuxR solos in fluorescent pseudomonads even could be classified into different subgroups based on their neighborhood genes and primary structure (36). Consequently, the 5-kbp genomic context flanking each LuxI/R homolog of acidophiles was investigated (Fig. 6). Three conserved genes always flanked LuxR homologs of *A. ferrooxidans*, (i) encoding a C4-dicarboxylate transporter, (ii) encoding a LysR family transcriptional regulator, and (iii) encoding a 3,4-dihydroxy-2-butanone 4-phosphate synthase (DHBPS). The C4-dicarboxylate transporter participates in the transport and metabolic processes of C4-dicarboxylate, a critical carbon source for bacteria. DHBPS is one of the key enzymes involved in the biosynthesis of riboflavin, which is essential for bacterial survival and proliferation. On the LuxI homolog side, an efflux ABC transporter, ATP-binding protein, a GNAT family acetyltransferase, an isonitrile hydratase, and an FMN-binding negative transcriptional regulator were presented but they were lost in strain BY-3 (Fig. S5). In addition, the LuxI homolog solo of BY-3 was flanked by genes coding for sulfur carrier protein FdhD, transposase, and 5-enolpyruvylshikimate-3-phosphate synthase. In view of the identification of the transposase, it is likely that the solo was transferred into the genome by transposon insertions (37). As for A. thiooxidans, it is worthy of note that one mobile element was present upstream of the LuxR homolog in most strains, such as ZBY (Fig. 6), implying former transposition events or transposition potential of the gene cluster (38). While the mobile element disappeared in strains BY-02 and JYC-17 (Fig. S5), and it was replaced by serine/threonine protein phosphatase and shufflon-specific DNA recombinase in strain CLST (Fig. 6). An acryloyl-CoA reductase was located adjacent to LuxI homologues in A. thiooxidans. A gene coding for transposase and inactivated derivatives was also identified in strain DMC (Fig. S5). The presence of functional genes of *A. ferridurans* and *Acidithiobacillus* sp. ‘AMD consortium’ was similar to that of *A. ferrooxidans*, and the adjacent loci also consisted of ribosomal protein S4 and related proteins, two transcriptional regulators of the AcrR and MarR families, and the 4-carboxymuconolactone decarboxylase domain (Fig. 6). In the LuxI homolog solo of *Acidiphilium* sp. 37-67-22, three genes encoding VirB1, VirB2 VirB4, which are related to the type IV secretion complex and T-DNA transfer were placed on the neighboring site. Lastly, except for the regulatory proteins, the proximal components of the LuxR homolog in L. ferrooxidans C2-3 were tautomerase, integrase on one side and dihydroflavonol-4-reductase, *N*-acetylornithine aminotransferase on the other side. Prior studies have shown that the adjacent proline iminopeptidase (*pip*) gene is under the nearby LuxR solo’s control in *Xanthomonas* plant pathogens and plant-beneficial Pseudomonas spp. (36). A basic picture of genome arrangement of LuxI/R homologues and their neighbors is provided in the current study, yet the probable interactions and effects are still to be resolved based on experimental means.

10.1128/msystems.01491-21.5 FIG S5 The arrangement of identified LuxI and LuxR homologs and the 5-kbp genetic region surrounding the selected acidophiles. Genes and their orientations are depicted with arrows using the following colors: red, LuxI homolog; yellow, LuxR homolog; gray, hypothetical protein. Download FIG S5, TIF file, 0.7 MB. Copyright © 2022 Huang et al. 2022 Huang et al. https://creativecommons.org/licenses/by/4.0/ This content is distributed under the terms of the Creative Commons Attribution 4.0 International license.

## DISCUSSION

**FIG 7:**
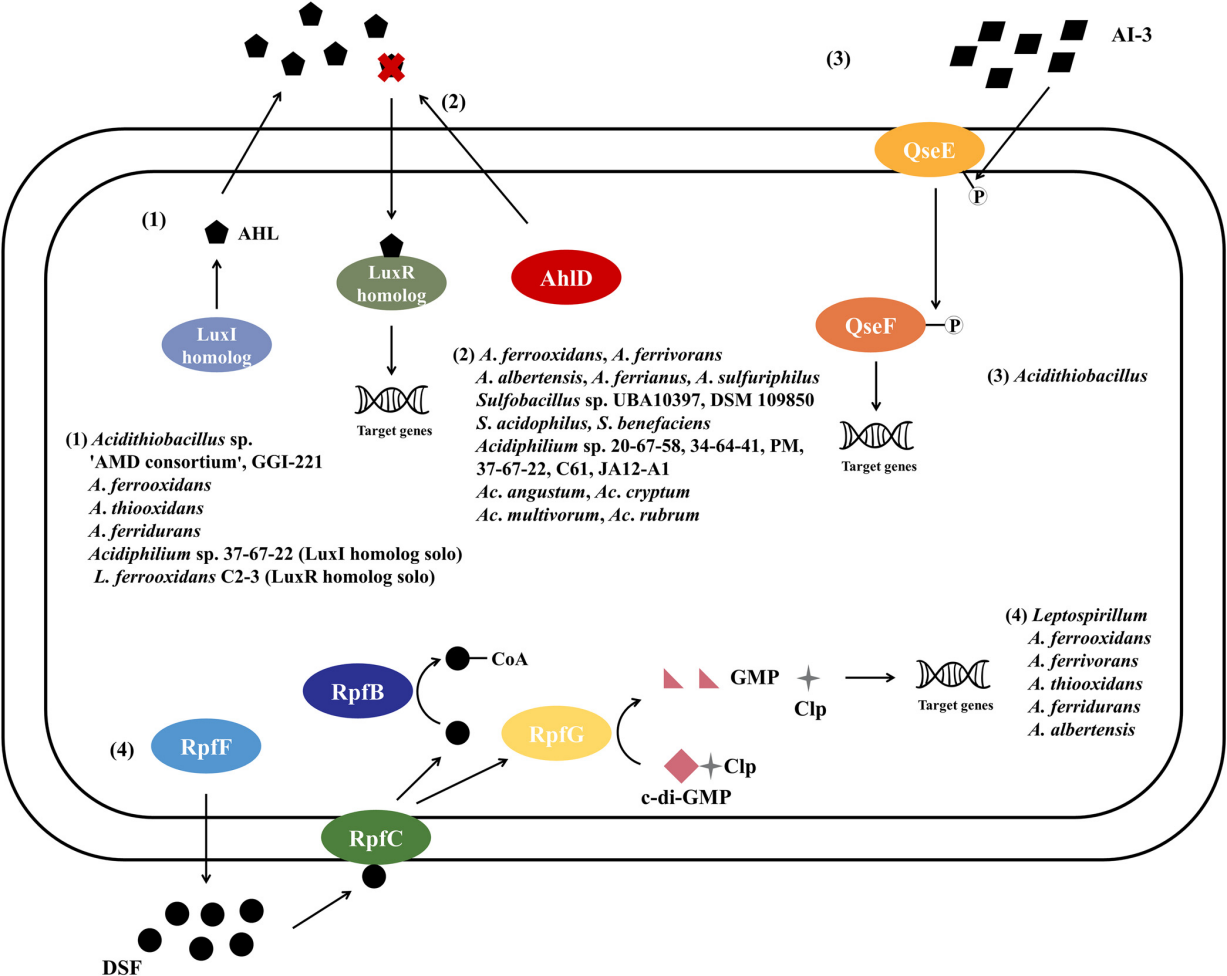
Overview of proposed QS system and QQ enzyme distribution in acidophiles. (1) AI-1 QS system; (2) AHL degradation enzyme of QQ; (3) QseE/F of AI-3 QS system; (4) DSF-QS system.

QS is an intercellular communication mechanism which enables bacteria to feel surrounding cues and coordinately adjust their density and behavior. It has been reported that some acidophilic bacteria have utilized this novel strategy to coordinate EPS synthesis, energy metabolism, or biofilm formation to control bioleaching activity and environmental adaptation (24, 39, 40). Here, a bioinformatic survey has been conducted to predict the existence of a QS system in some acidophiles at the genomic level. Then, the conservation, probable function, and evolutionary aspects of QS-related proteins were explored. A schematic distribution of QS/QQ systems among acidophiles was concluded (Fig. 7). *Acidithiobacillus* strains encoded three types of QS system and one QQ type of AHL degradation enzyme. Some individuals of *Sulfobacillus* and *Acidiphilium* have evolved an enzyme for AHL inactivation, potentially playing crucial roles against other competitors. An effective DSF-type signaling system was notably specific to *Leptospirillum* in particular.

### AHL molecules cleaved by acidophiles with(out) AHL production capacity for effective niche exploitation.

The AI-1 system is one major QS type which has been characterized widely. Meanwhile, the related LuxI/LuxR and their homologues are focused on and examined extensively via *in silico* analyses (41, 42). Using the domain-based strategy and key residue comparison, we found that an adjacent LuxI/R homolog pair was carried by most *A. ferrooxidans* and A. thiooxidans strains, similar to the distribution of the AfeI/AfeR system based on sequence BLAST analysis (22). The AfeI/R (LuxI/R like) system has been defined and verified in *A. ferrooxidans*, which could intelligently drive energy metabolism, cell growth, and EPS secretion in Fe^2+^/S^0^-enriched medium to benefit themselves (22). Nine different chemical AHL molecules with diverse C-3 substitutions (hydroxyl and oxo) and only even numbers (between 8 and 16) of carbons in the acyl chain (3-hydroxy-C_8_-AHL, 3-hydroxy-C_10_-AHL, C_12_-AHL, 3-oxo-C_12_-AHL, 3-hydroxy-C_12_-AHL, C_14_-AHL, 3-oxo-C_14_-AHL, 3-hydroxy-C_14_-AHL, and 3-hydroxy-C_16_-AHL) are detected from *A. ferrooxidans* ATCC 23270 cultures (21). Plentiful 3-hydroxy-C_14_-AHL is traced in Fe^2+^- and S^0^-enriched media, which could stimulate regulation of EPS synthesis and cell growth in S^0^-enriched medium but not work in Fe^2+^-enriched medium (22). In addition, for the two kinds of 3-oxo-AHL compounds with 12 or 14 carbons in the large acyl chains, they are produced only by sulfur- and thiosulfate-grown cells, and the function effects are still to be characterized (21). It is common that AHL synthases are able to produce more than one kind of AHL. The SinI of Sinorhizobium meliloti synthesizes five different forms of AHLs, ranging from C_12_-AHL to C_18_-AHL, including some oxo-AHLs and a monounsaturated AHL (43). Longer-chain AHLs seem to be more insulated from chemical degradation and utilized by microorganisms in harsher environments, suggesting that cross talk might emerge between *A. ferrooxidans* and other bacterial species inhabiting the bioleaching ecological niche (44). According to the evolutionary relatedness, LuxI and LuxR homolog proteins have similar clustering characteristics, indicating that they may be coevolving and cofunctioning (45). The tree clades clustered mainly in accordance to the species taxonomy, and the AI-1 system of *A. ferrooxidans* and A. thiooxidans exhibited a clear division. In contrast, the system showcased a relatively higher degree of evolutionary relatedness within the same species. Moreover, the LuxI homolog solo of *Acidiphilium* sp. 37-67-22 was evolutionarily distant and shared similar crystal structures and vital substitution of a mutant RhlI, suggesting its authenticity and functionality in AHL with 3-oxo group synthesis. Though RhlI is reported to mainly produce *N*-butanoyl (BHL) and *N*-hexanoyl (HHL) homoserine lactones, which are AHLs without 3-oxo groups (31). Chemical communication between *Acidiphilium* strains and *Acidithrix* strains has effectively enhanced growth and Fe(II) oxidation rates. The mediated QS molecules still need in-depth inspection, then (46). The LuxR homolog solo in L. ferrooxidans C2-3 has also been confirmed, and the same critical residues have been identified in a bioinformatic survey, clustering with LuxR solos of Methylacidiphilum fumariolicum, Methylacidiphilum infernorum, and Nitrospira defluvii in the phylogenetic tree (47). The phenomenon of biofilm formation triggered by QS has been observed through a transcriptome technique (48); we thus postulated that its LuxR solo could sense AI-1 produced by other bacteria such as *A. ferrooxidans*, which always live with them in extremely acidic waters, and then modulate relevant gene expression. As a dominant organism in AMD environments, *A. ferrooxidans* has all kinds of the above-mentioned communication tools, which may provide strong assistance for its desirable adaptation.

Since AHL-dependent signaling strategies are widespread and attractive among bacteria, a signal interference method that disrupted the QS system has come into view over the past decade. The AHL-degradation enzyme AhlD in *Arthrobacter* sp. has been discussed and predicted to exist in some other bacteria (32). The AhlD distribution spanned various species of *Acidithiobacillus*, *Sulfobacillus*, and *Acidiphilium* and harbored a conserved motif, HXHXDH≈H≈D, necessary for enzymatic activity. Interestingly, except for *A. ferrooxidans* BY-3 and *Acidiphilium* sp. 37-67-22, the others did not possess any AHL-QS system-linked proteins. Some bacteria could cleave their own AHL signal, such as *Agrobacterium* and Pseudomonas (13). Owing to extensive QQ activities within the phyla *Proteobacteria*, *Bacteroidetes*, *Actinobacteria*, and *Firmicutes*, it is likely that many acidophiles may fight against others with AHL-emitting capacity or balance the amount of AHL produced by themselves for efficient resource and niche utilization by this approach (13).

### DSF combined with c-di-GMP to jointly regulate biofilm formation among acidophiles.

The DSF-based QS system represents an intriguing type of cell-to-cell communication mechanism in diverse G^–^ bacteria. Multiomics and genetic methods have unveiled a complete DSF system possessed by L. ferriphilum DSM 14647^T^ (49) and a similar *rpf* gene cluster with a complete RpfC homologue contained in L. ferrooxidans C2-3 (50). As well as in *Leptospirillum* species, we also found the DSF type QS carried by several *A. ferrivorans* and A. thiooxidans strains, *A. ferrooxidans* BY0502, *A. ferridurans* IO-2C, and *A. albertensis* DSM 14366. By sequence alignments, signature residues or motifs were screened in RpfF essential for DSF biosynthesis, and RpfB engaged in the turnover of the DSF family signals. The signaling sensor RpfC and transduction RpfG were distributed in terms of taxonomic lineages and displayed similar evolutionary patterns in the phylogenetic tree. Several previously described studies have discussed the vital biological effects of DSF family compounds in *Leptospirillum*. After adding DSF, the amounts of L. ferriphilum and *S. thermosulfidooxidans* adhering on minerals decrease, leading to biofilm dispersal and preventing the formation of a passivation layer, which is essential for the bioleaching performance (51). Moreover, when DSF production by L. ferriphilum microcolonies is prior to the addition of *S. thermosulfidooxidans*, DSF molecules specially suppress Fe^2+^ oxidation of exogenous *S. thermosulfidooxidans* cells, thereby making the energy resource available specifically to the DSF releaser (52). Here, the *Leptospirillum* sp. DSF system may be an efficient niche protection strategy and could resist against other unfavored biomining populations. QS and c-di-GMP signaling system are considered to be the primary methods modulating biofilm formation and EPS production in G^–^ acidophiles (53). The c-di-GMP metabolism elements have been predicted and compared in A. thiooxidans, *A. ferrivorans*, *A. ferrooxidans*, *A. caldus*, and *A. albertensis* (54). Meanwhile, its functions in adjusting motility and adherence have been confirmed in several *Acidithiobacillus* and *Leptospirillum* species (24, 55–57). It has been acknowledged that the activated RpfG has phosphodiesterase ability and could degrade c-di-GMP, the congenital ligand of the transcription factor Clp. Ultimately, derepressed Clp alters the expression level of abundant genes, such as those coding virulence factors (58). There is reason to expect that the DSF integrated with c-di-GMP pathways plays pivotal roles in population communication and adaptation, just like the connection between AHL-mediated QS and c-di-GMP during the process of colonization and dissolution of minerals (4, 59).

**Concluding remarks.** In summary, our analysis provided a picture of the distribution, phylogeny, and putative functions of QS/QQ-related proteins among acidophiles belonging to four genera. The presence and authenticity of QS systems and QQ enzymes were emphasized, which played a critical role in establishing communication circuits or disturbing signal propagation for valuable niche exploitation. Intra- or interspecies contact could occur via “dialects” in the acidophilic microbe world, opening new perspectives for the regulatory networks of gene expression and adaptive evolution. More experiments are needed to investigate the ecological functions of QS/QQ in microbial communities among acidophiles.

## MATERIALS AND METHODS

### Genome data retrieval and annotation.

The genomic sequences of the acidophile genomes were downloaded from the NCBI (https://www.ncbi.nlm.nih.gov/) website. Of those, 41 were affiliated with the *Acidithiobacillus* genus, 12 were *Leptospirillum*, 17 were *Sulfobacillus*, and 13 were *Acidiphilium*. The protein sequences were annotated by BLAST KOALA (KEGG Orthology and Links Annotation) (60) for KO (KEGG Orthology) assignment, which was performed by the KOALA algorithm using the weighted sum of BLAST bit scores. Based on the functional classification of “cellular community-prokaryotes,” the information of QS-associated proteins in each strain was investigated at “quorum sensing” (ko02024) pathways. In addition, genomic sequences were submitted to the RAST (Rapid Annotations using Subsystems Technology) (61) server to identify QS-related proteins. These candidate proteins were then submitted to a BLAST search against the Cluster of Orthologous Groups of proteins (COGs) database to assign function annotations. The presence and components of the adjacent regions around them were also obtained.

### Systematic bioinformatics prediction of LuxI and LuxR homologs.

For the stringent identification of putative LuxI/R homologs, a profile Hidden Markov model (HMM)-based similarity search (E value, <1e-5) was conducted to scan for the signature protein family (Pfam) domain contained in reported LuxI (PF00765, autoinducer synthase) and LuxR (PF03472, autoinducer-binding domain), respectively. In addition, three signature domains, IPR016181 (acyl-CoA N-acyltransferase), IPR001690 (autoinducer synthase), and IPR018311 (autoinducer synthesis, conserved site), are universally present in the functionally validated LuxIs, while a canonical LuxR always contains four signature domains, IPR005143 (autoinducer-binding domain), IPR036388 (winged helix-turn-helix DNA-binding domain), IPR016032 (signal transduction response regulator, C-terminal effector), and IPR000792 (transcription regulator LuxR, C-terminal) (41). To further verify the high confidence of LuxI/R homologs, the shortlisted proteins were subsequently subjected to InterProScan 5 (62) for conserved domain architecture searching.

### Multiple sequence alignment (MSA) and phylogenetic analyses.

The LuxI/R homolog proteins (RhlI, KO number K13061; RaiI, K20249; SolR, K19666) of AI-1 QS system, proteins (RpfB, K01897; RpfF, K13816) of DSF-QS system, and a protein (AhlD, K13075) of QQ system sequences found in this study were aligned using Clustal Omega to check the sequence similarity and visualize the signature conserved residues (63). The default parameters used were as follows: Gonnet transition matrix, 6-bits gap opening penalty with 1-bit gap extension.

Evolutionary analyses were carried out using MEGA X (64). Protein sequences of the AI-1 QS system (RhlI, RaiI, and SolR), AI-3 QS system (QseE, KO number K07711; QseF, K07715), and DSF-QS system (RpfC, K10715; RpfG, K13815) were aligned with Clustal W (65). Further, the phylogenetic trees were constructed employing the neighbor-joining (NJ) method based on the Poisson model, which generated trees with uniform rates and evaluated them with a bootstrap value of 5,000.

### Molecular modeling of proteins.

The three-dimensional (3D) model of the RaiI protein entry from *Acidiphilium* sp. 37-67-22 was generated by the automatic protein homology-modeling tool SWISS-MODEL (Swiss Institute of Bioinformatics) (66). The template used was the crystal structure of the related enzyme CoA-dependent acyl-homoserine lactone synthase, RpaI, from Rhodopseudomonas palustris (PDB: 6wn0) with 27.32% sequence identity at 1.80 Å resolution. The quality of the model was satisfactory with a QMEAN score in the range of 0.63 and 0.75.
