# Supplementary material for: Insights into Adaptive Mechanisms of Extreme Acidophiles Based on Quorum Sensing/Quenching-Related Proteins
Source: mSystems. 2022 Apr 11;7(2):e01491-21. doi: 10.1128/msystems.01491-21 (PMC9040811; doi:10.1128/msystems.01491-21)
Supplement: TABLE S1 [file msystems.01491-21-s0006.docx]

**Table S1.** Main features and QS-related annotation of the 83 acidophiles.

| **Strain** | **GenBank assembly** | **Level** | **Size (Mb)** | **GC%** | **No. of annotated entries** | **No. of annotated**  **KO categories** |
| --- | --- | --- | --- | --- | --- | --- |
| *Acidithiobacilllus caldus* ATCC 51756 | GCA_000175575.2 | complete | 2.99 | 61.18 | 31 | 28 |
| *Acidithiobacilllus caldus* DX | GCA_001756675.1 | contig | 3.12 | 61.00 | 37 | 28 |
| *Acidithiobacilllus caldus* MTH-04 | GCA_001650235.3 | complete | 2.95 | 61.39 | 29 | 27 |
| *Acidithiobacillus caldus* S1 | GCA_001756775.1 | contig | 2.79 | 60.90 | 21 | 20 |
| *Acidithiobacillus caldus* SM-1 | GCA_000221025.1 | complete | 3.24 | 60.94 | 40 | 28 |
| *Acidithiobacillus caldus* ZBY | GCA_001756725.1 | contig | 3.16 | 61.00 | 37 | 28 |
| *Acidithiobacillus caldus* ZJ | GCA_001756745.1 | contig | 3.14 | 61.00 | 37 | 28 |
| *Acidithiobacillus ferrivorans* 21-59-9 | GCA_002255305.1 | scaffold | 2.70 | 59.00 | 34 | 22 |
| *Acidithiobacillus ferrivorans* CF27 | GCA_000750615.1 | contig | 3.43 | 56.40 | 36 | 32 |
| *Acidithiobacillus ferrivorans* PQ33 | GCA_001857665.2 | contig | 3.32 | 56.61 | 37 | 34 |
| *Acidithiobacillus ferrivorans* PRJEB5721 | GCA_900174455.1 | chromosome | 3.46 | 56.51 | 36 | 32 |
| *Acidithiobacillus ferrivorans* SS3 | GCA_000214095.3 | complete | 3.21 | 56.60 | 35 | 33 |
| *Acidithiobacillus ferrivorans* XJFY6S-08 | GCA_016250455.1 | complete | 3.16 | 56.50 | 49 | 37 |
| *Acidithiobacillus ferrivorans* YL15 | GCA_001685225.1 | contig | 3.00 | 56.60 | 39 | 35 |
| *Acidithiobacillus ferrooxidans* ATCC 23270 | GCA_000021485.1 | complete | 2.98 | 58.80 | 38 | 34 |
| *Acidithiobacillus ferrooxidans* ATCC 53993 | GCA_000020825.1 | complete | 2.89 | 58.90 | 47 | 34 |
| *Acidithiobacillus ferrooxidans* BY0502 | GCA_001652185.1 | contig | 2.98 | 56.80 | 27 | 22 |
| *Acidithiobacillus ferrooxidans* BY-3 | GCA_010577825.1 | contig | 3.83 | 57.80 | 45 | 36 |
| *Acidithiobacillus ferrooxidans* CCM 4253 | GCA_003233765.1 | contig | 3.20 | 58.60 | 70 | 36 |
| *Acidithiobacillus ferrooxidans* Hel18 | GCA_001559335.1 | contig | 3.11 | 58.60 | 66 | 36 |
| *Acidithiobacillus ferrooxidans* RVS1 | GCA_003931975.1 | contig | 2.83 | 58.80 | 41 | 33 |
| *Acidithiobacillus ferrooxidans* YNTRS-40 | GCA_013462805.1 | complete | 3.26 | 58.47 | 66 | 36 |
| *Acidithiobacillus ferrooxidans* YQH-1 | GCA_001418795.1 | scaffold | 3.11 | 58.60 | 66 | 36 |
| *Acidithiobacillus thiooxidans* A01 | GCA_000559045.1 | contig | 3.82 | 53.10 | 70 | 37 |
| *Acidithiobacillus thiooxidans* A02 | GCA_001705645.1 | contig | 3.72 | 53.00 | 74 | 39 |
| *Acidithiobacillus thiooxidans* ATCC 19377 | GCA_009662475.1 | complete | 3.42 | 53.00 | 48 | 29 |
| *Acidithiobacillus thiooxidans* BY-02 | GCA_001705725.1 | contig | 3.81 | 53.10 | 72 | 39 |
| *Acidithiobacillus thiooxidans* CLST | GCA_002079865.1 | scaffold | 3.97 | 52.40 | 59 | 32 |
| *Acidithiobacillus thiooxidans* DMC | GCA_001705625.1 | contig | 3.85 | 53.10 | 75 | 39 |
| *Acidithiobacillus thiooxidans* DXS-W | GCA_001705805.1 | contig | 3.95 | 52.90 | 70 | 39 |
| *Acidithiobacillus thiooxidans* GD1-3 | GCA_001705695.1 | contig | 3.95 | 52.90 | 69 | 39 |
| *Acidithiobacillus thiooxidans* JYC-17 | GCA_001705755.1 | contig | 3.83 | 53.10 | 76 | 40 |
| *Acidithiobacillus thiooxidans* Licanantay | GCA_000709715.1 | contig | 3.94 | 52.80 | 47 | 29 |
| *Acidithiobacillus thiooxidans* ZBY | GCA_001756595.1 | contig | 3.79 | 53.20 | 74 | 38 |
| *Acidithiobacillus ferridurans* IO-2C | GCA_003309025.1 | contig | 2.72 | 58.70 | 35 | 32 |
| *Acidithiobacillus ferridurans* JCM 18981 | GCA_003966655.1 | complete | 2.92 | 58.40 | 60 | 37 |
| *Acidithiobacillus albertensis* DSM 14366 | GCA_001931655.1 | scaffold | 3.50 | 52.60 | 43 | 33 |
| *Acidithiobacillus ferrianus* MG | GCA_010378095.1 | contig | 3.17 | 58.20 | 30 | 25 |
| *Acidithiobacillus sulfuriphilus* CJ-2 | GCA_003721225.1 | contig | 2.82 | 61.50 | 25 | 22 |
| *Acidithiobacillus* sp. 'AMD consortium' | GCA_008926505.1 | complete | 2.93 | 58.60 | 38 | 34 |
| *Acidithiobacillus* sp. GGI-221 | GCA_000179815.2 | scaffold | 3.17 | 58.60 | 53 | 29 |
| *Leptospirillum ferriphilum* ZJ | GCA_002002665.1 | contig | 2.34 | 54.70 | 23 | 22 |
| *Leptospirillum ferriphilum* YSK | GCA_000695975.1 | complete | 2.33 | 54.50 | 23 | 22 |
| *Leptospirillum ferriphilum* SpSt-902 | GCA_011332985.1 | contig | 2.14 | 54.60 | 28 | 25 |
| *Leptospirillum ferriphilum* Sp-Cl | GCA_001280545.1 | contig | 2.48 | 54.40 | 23 | 22 |
| *Leptospirillum ferriphilum* pb_238 | GCA_900198525.1 | contig | 2.61 | 54.10 | 32 | 30 |
| *Leptospirillum ferriphilum* ML-04 | GCA_000299235.1 | complete | 2.41 | 54.60 | 23 | 22 |
| *Leptospirillum ferriphilum* DX | GCA_002002505.1 | contig | 2.36 | 54.50 | 23 | 22 |
| *Leptospirillum ferriphilum* DSM 14647 | GCA_000755505.1 | contig | 2.41 | 54.10 | 31 | 29 |
| *Leptospirillum rubarum* LeptoII | GCA_000205145.2 | scaffold | 2.64 | 54.70 | 30 | 29 |
| *Leptospirillum ferrooxidans* C2-3 | GCA_000284315.1 | complete | 2.56 | 50.00 | 24 | 24 |
| *Leptospirillum* sp. Group II 'CF-1' | GCA_001186405.1 | complete | 2.71 | 54.60 | 23 | 22 |
| *Leptospirillum* sp. Group II 'C75' | GCA_000262365.1 | scaffold | 2.61 | 54.40 | 23 | 22 |
| *Sulfobacillus thermosulfidooxidans* ZJ | GCA_001953295.1 | contig | 3.18 | 48.50 | 59 | 32 |
| *Sulfobacillus thermosulfidooxidans* ZBY | GCA_001953275.1 | contig | 3.18 | 48.50 | 59 | 32 |
| *Sulfobacillus thermosulfidooxidans* ST | GCA_000497695.1 | scaffold | 3.33 | 48.30 | 65 | 32 |
| *Sulfobacillus thermosulfidooxidans* DX | GCA_001953285.1 | contig | 3.18 | 48.50 | 59 | 32 |
| *Sulfobacillus thermosulfidooxidans* DSM 9293 | GCA_900176145.1 | scaffold | 3.86 | 49.70 | 61 | 32 |
| *Sulfobacillus thermosulfidooxidans* Cutipay | GCA_000294425.1 | scaffold | 3.86 | 49.30 | 59 | 32 |
| *Sulfobacillus thermosulfidooxidans* CBAR-13 | GCA_001280565.1 | scaffold | 3.83 | 48.90 | 54 | 31 |
| *Sulfobacillus thermosulfidooxidans* AMDSBA5 | GCA_003023625.1 | scaffold | 3.65 | 49.10 | 53 | 30 |
| *Sulfobacillus acidophilus* TPY | GCA_000219855.1 | complete | 3.55 | 56.80 | 54 | 34 |
| *Sulfobacillus acidophilus* DSM 10332 | GCA_000237975.1 | complete | 3.56 | 56.79 | 54 | 34 |
| *Sulfobacillus acidophilus* AMDSBA3 | GCA_003023745.1 | scaffold | 3.68 | 55.50 | 70 | 24 |
| *Sulfobacillus benefaciens* AMDSBA4 | GCA_003023695.1 | scaffold | 4.11 | 51.60 | 70 | 33 |
| *Sulfobacillus benefaciens* AMDSBA1 | GCA_003023725.1 | scaffold | 4.56 | 52.20 | 66 | 32 |
| *Sulfobacillus thermotolerans* Kr1 | GCA_002951815.1 | complete | 3.32 | 52.40 | 65 | 31 |
| *Sulfobacillus* sp. UBA10397 | GCA_003511545.1 | scaffold | 3.29 | 51.20 | 29 | 19 |
| *Sulfobacillus* sp. hq2 | GCA_002903155.1 | scaffold | 3.43 | 52.60 | 68 | 34 |
| *Sulfobacillus* sp. DSM 109850 | GCA_012933365.1 | contig | 4.40 | 58.20 | 89 | 35 |
| *Acidiphilium rubrum* ATCC 35905 | GCA_900156265.1 | scaffold | 3.98 | 63.70 | 96 | 40 |
| *Acidiphilium multivorum* AIU301 | GCA_000202835.1 | complete | 4.21 | 66.99 | 94 | 46 |
| *Acidiphilium cryptum* JF-5 | GCA_000016725.1 | complete | 3.96 | 67.10 | 85 | 37 |
| *Acidiphilium angustum* ATCC 35903 | GCA_000701585.1 | scaffold | 4.18 | 63.60 | 96 | 40 |
| *Acidiphilium* sp. PM | GCA_000219295.2 | scaffold | 3.93 | 66.40 | 100 | 47 |
| *Acidiphilium* sp. JA12-A1 | GCA_000724705.2 | contig | 4.18 | 66.90 | 87 | 35 |
| *Acidiphilium* sp. C61 | GCA_902712915.1 | contig | 3.85 | 66.10 | 72 | 40 |
| *Acidiphilium* sp. 37-67-22 | GCA_002279335.1 | scaffold | 2.48 | 67.00 | 56 | 27 |
| *Acidiphilium* sp. 37-60-79 | GCA_002279355.1 | contig | 3.07 | 60.00 | 69 | 31 |
| *Acidiphilium* sp. 34-64-41 | GCA_002282635.1 | scaffold | 3.86 | 63.70 | 86 | 36 |
| *Acidiphilium* sp. 34-60-192 | GCA_002282645.1 | scaffold | 3.11 | 60.10 | 60 | 29 |
| *Acidiphilium* sp. 21-60-14 | GCA_002255745.1 | contig | 3.06 | 60.20 | 75 | 37 |
| *Acidiphilium* sp. 20-67-58 | GCA_002255515.1 | contig | 3.41 | 66.60 | 59 | 31 |
