# Supplementary figures and images for: Insights into Adaptive Mechanisms of Extreme Acidophiles Based on Quorum Sensing/Quenching-Related Proteins
Source: mSystems. 2022 Apr 11;7(2):e01491-21. doi: 10.1128/msystems.01491-21 (PMC9040811; doi:10.1128/msystems.01491-21)

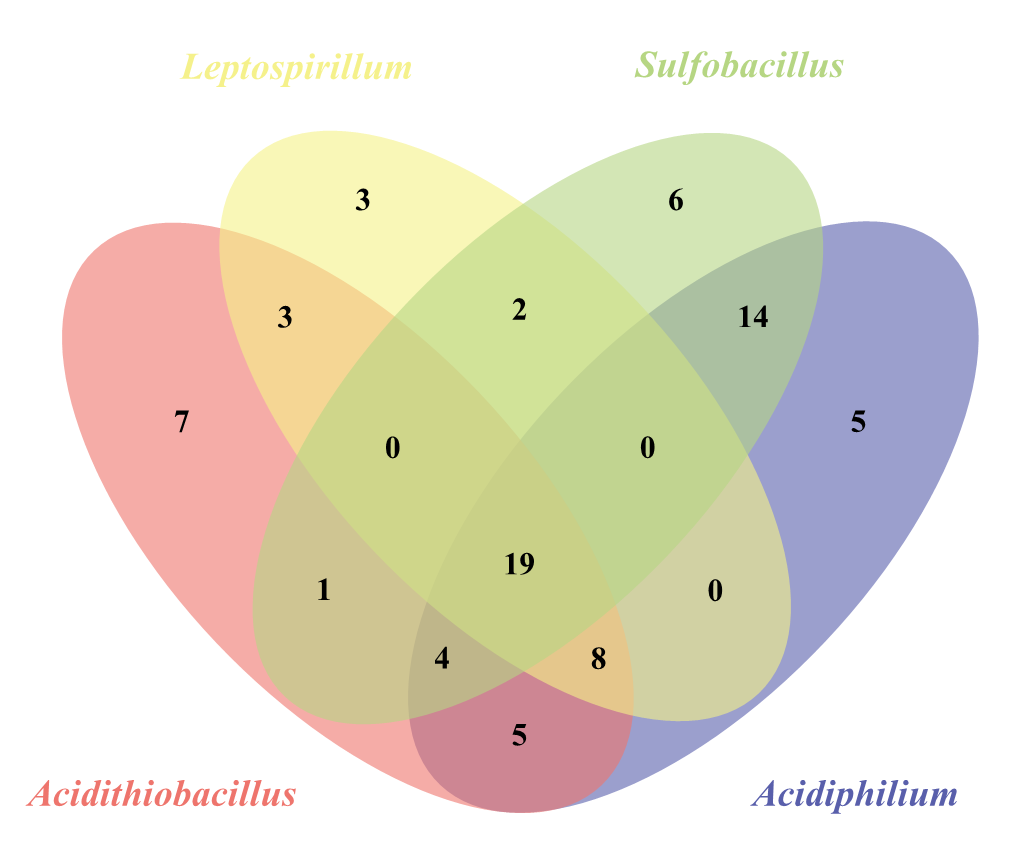

Supplement: FIG S1 [file msystems.01491-21-s0001.tif]

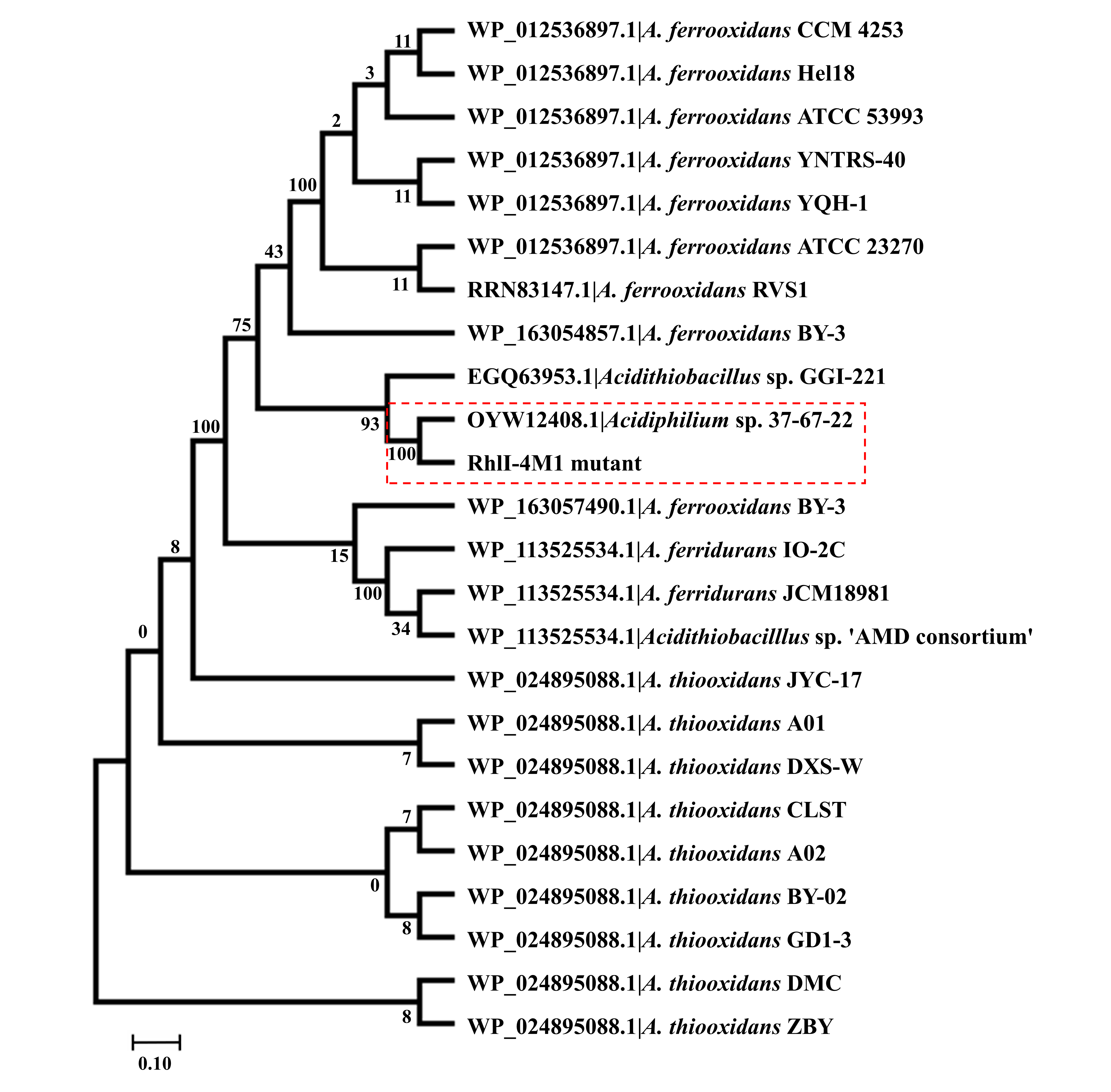

Supplement: FIG S2 [file msystems.01491-21-s0002.tif]

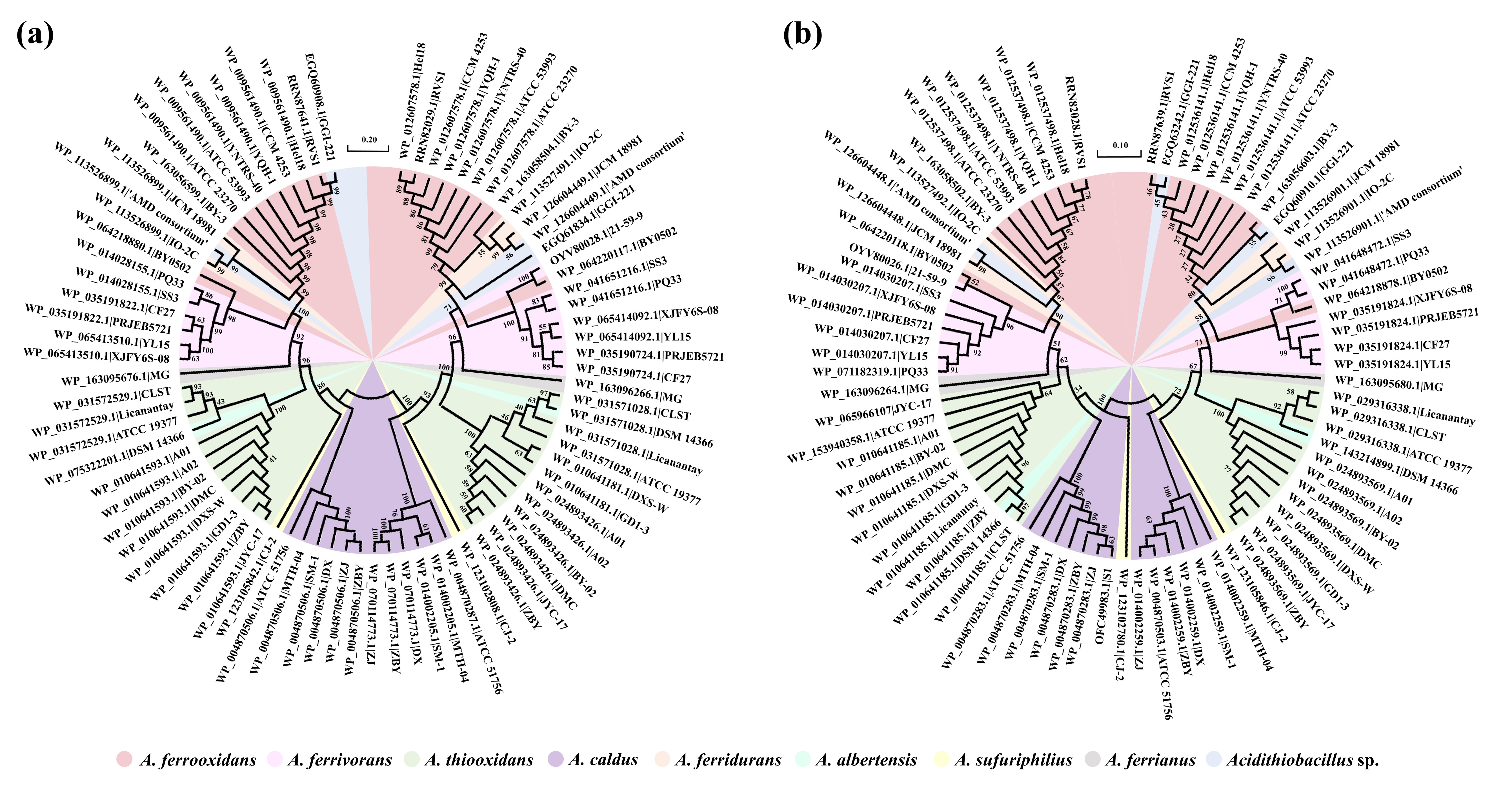

Supplement: FIG S3 [file msystems.01491-21-s0003.tif]

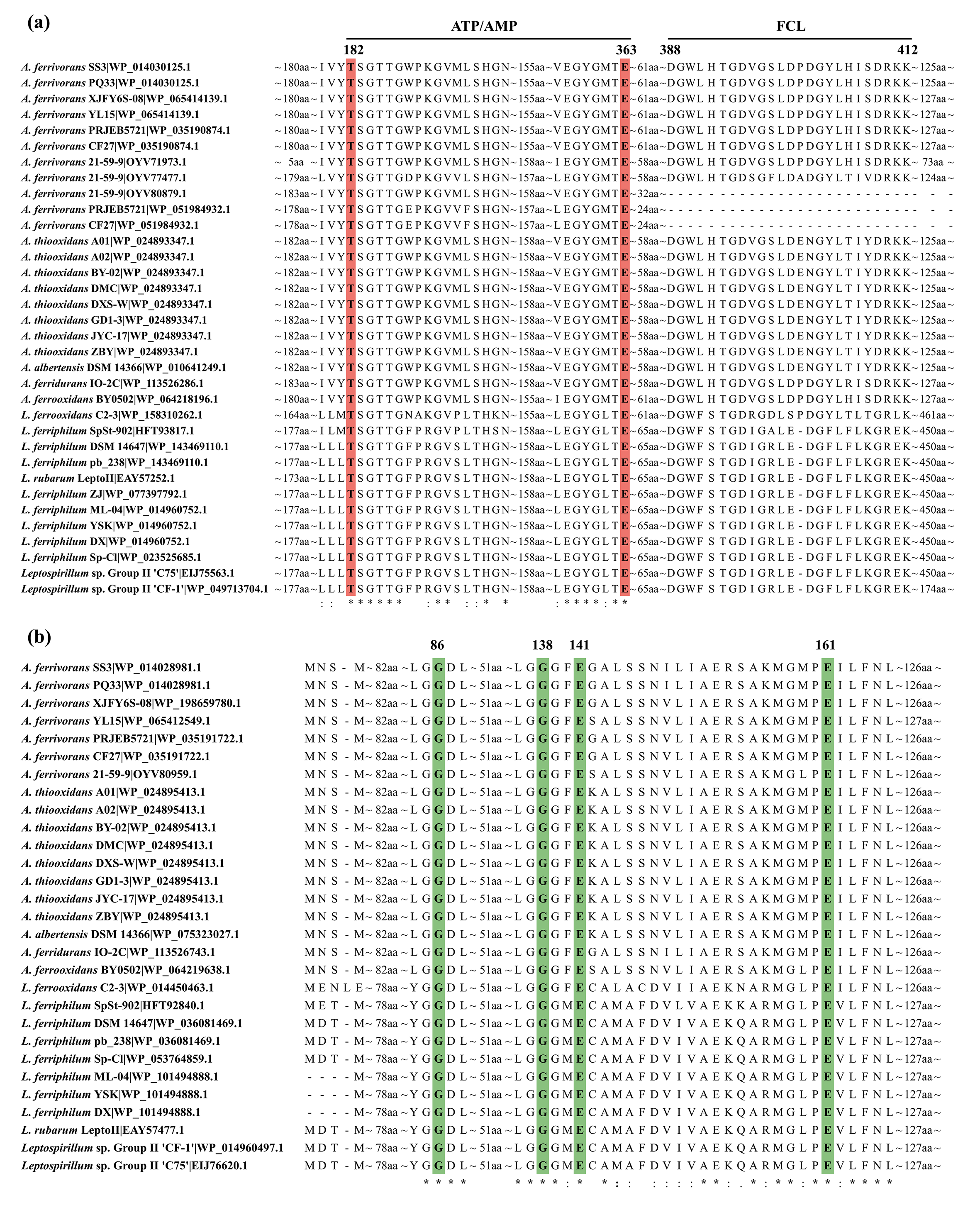

Supplement: FIG S4 [file msystems.01491-21-s0004.tif]

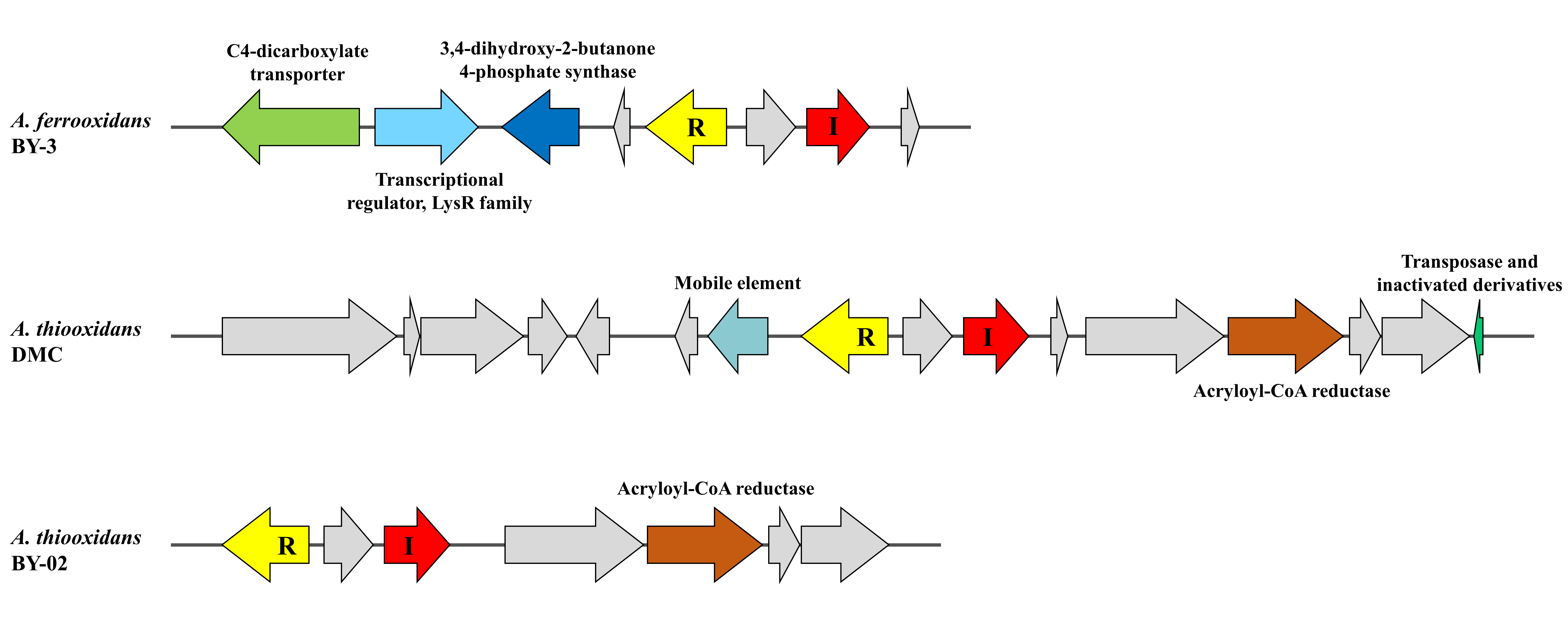

Supplement: FIG S5 [file msystems.01491-21-s0005.tif]
